# Pharmacological Upregulation of Microglial Lipid Droplet Alleviates Neuroinflammation and Acute Ischemic Brain Injury

**DOI:** 10.1007/s10753-023-01844-z

**Published:** 2023-07-14

**Authors:** Huiya Li, Pinyi Liu, Shiji Deng, Liwen Zhu, Xiang Cao, Xinyu Bao, Shengnan Xia, Yun Xu, Bing Zhang

**Affiliations:** 1 Department of Radiology, Nanjing Drum Tower Hospital, Affiliated Hospital of Medical School, Nanjing University, Nanjing, 210008 China; 2 Department of Neurology, Nanjing Drum Tower Hospital, Affiliated Hospital of Medical School, Nanjing University, Nanjing, 210008 China; 3 Institute of Medical Imaging and Artificial Intelligence, Nanjing University, Nanjing, 210008 China; 4 Medical Imaging Center, Nanjing Drum Tower Hospital, Affiliated Hospital of Medical School, Nanjing University, Nanjing, 210008 China; 5 Jiangsu Key Laboratory of Molecular Medicine, Nanjing, 210008 China; 6 Institute of Brain Science, Nanjing University, Nanjing, 210008 China

**Keywords:** Lipid droplets, Ischemic stroke, Microglia, Atglistatin

## Abstract

Lipid droplets (LDs) were reported to play an important role in the modulation of inflammation and various cellular processes among multiple cell types. However, LDs accumulation, its function and mechanisms of its formation during ischemic stroke remained poorly-identified. In this study, we observed increased LDs accumulation in microglia at the acute stage of ischemic stroke by immunofluorescence and flow cytometry. Transcriptomic analysis indicated that microglia accumulated with LDs were associated with inflammation and phagocytosis. Both inflammatory activation and phagocytosis of tissue debris in microglia could contribute to LDs formation. Moreover, through specific LDs depletion and overload experiments by pharmacological approaches, we proposed that LDs was critical for the maintenance of anti-inflammatory properties of microglia. Furthermore, Atglistatin, a specific adipose triglyceride lipase (ATGL) inhibitor, was shown to prevent proinflammatory cytokines production in primary microglia through decreased LDs lipolysis. After Atglistatin treatment, middle cerebral artery occlusion (MCAO) mice showed decreased infarct volume and improved neurobehavioral performance at the acute stage of stroke. Our findings provided a biological basis for microglial LDs regulation as a potential therapeutic strategy for acute ischemic stroke and uncovered the neuroprotective role of Atglistatin in the treatment of MCAO mice.

**Supplementary Information:**

The online version contains supplementary material available at 10.1007/s10753-023-01844-z.

## INTRODUCTION

Lipid droplet (LD) was an organelle containing neutral lipid such as triacylglycerols and cholesteryl esters, which was surrounded by a phospholipid monolayer [1]. The hydrophobic core comprises the esterification of activated fatty acids while the enclosing monolayer consisted of specific sets of proteins. Originating from the endoplasmic reticulum (ER), LD was extremely sensitive to various stimulus [2]. Pattern recognition receptors (PRRs) were reported to be pivotal signaling mediating LDs accumulation within immune cells, especially the TLR family. As a TLR4 ligand, lipopolysaccharide (LPS) promoted the formation of LDs both *in vivo* and *in vitro* [1]. Reactive oxygen species (ROS) was also demonstrated to play a role in LDs accumulation among glial cells [3, 4]. If necessary, fatty acids stored in LDs could be mobilized through lipolysis or lipophagy to fuel metabolic processes or inflammatory mediators production [5]. The long-held view that LD was a storage organelle was no longer tenable. By sequestering excess lipids and releasing them based on cellular demands, LD has been recognized as a critical hub in the prevention of lipotoxicity, ER stress and mitochondrial damage as well as the modulation of inflammation [1, 2]. Either impaired fatty acids storage in LDs or dysregulated lipids catabolism of LDs could lead to the onset of diseases [2, 5].

Microglia, the resident immune cells in the central nervous system (CNS), surveyed the cerebral microenvironment consistently. After ischemia onset, microglia acted as rapid responders to initiate immune reactions. Various neural cells susceptible to ischemic insult gradually died and developed a large amount of cellular or myelin debris within the infarct, which were subsequently engulfed by microglia [6]. Reactive microglia also secreted multiple pro-inflammatory cytokines and chemokines, exacerbating inflammation-associated injuries. Thus, neuroinflammation mediated by microglia played a vital role in the progression and prognosis of ischemic stroke. However, whether inflammatory activation in microglia promotes LDs accumulation and how LDs influence microglial functions during ischemic stroke remain unclear.

LDs accumulation in microglia has been observed in the aging brain, demyelinating lesions, spinal cord injury (SCI), traumatic brain injury (TBI), Parkinson’s disease (PD) and Alzheimer’s disease (AD) [7–15]. Depending on different pathological microenvironments, LDs exerted distinct functions in microglia. In the aging brain, lipid-droplet-accumulating microglia displayed diminished phagocytosis capacity and enhanced pro-inflammatory properties [7–9]. Traumatic brain injury-induced LDs in microglia was correlated with the extent of microglial activation [12]. LDs induced by APOE4 led to impaired microglial surveillance of neuronal network [13]. Like foamy macrophage in the atherosclerosis, microglia with accumulated LDs were also called foamy microglia. Foamy microglia containing LDs, however, were regarded as a requirement for remyelination after demyelinating injury [10]. Thus, it is reasonable to conclude that LDs may influence microglial functions in a context-specific manner in response to different pathogenic insults. Our study revealed foamy microglia associated with ischemic stroke and the potential triggers for LDs accumulation in microglia. We also indicated an inflammation-alleviating role of foamy microglia during brain ischemia, providing promising new therapeutic strategies for stroke treatments.

## MATERIALS AND METHODS

### Cell Culture and *in Vitro* Experiments

Primary microglia were prepared from 1-day-old male C57/BL6J mice as previously described [16]. The cells were cultured in medium composed of Dulbecco’s Modified Eagle Medium (Invitrogen, Frederick, MD, USA), 10% fetal bovine serum (Hyclone, Logan, UT, USA) and 100 U/mL antibiotics, at 37 ℃ in a humid atmosphere containing 5% CO_2_. After culturing for 10–12 days, primary microglia were harvested by shaking for 5–10 min and replanted in new plates.

For *in vitro* experiments, LPS and cell debris were used to treat the primary microglia respectively. As for LPS-treated experiments, cells seeded in 12-well plates were divided into groups as follows: control group, DMSO-treated group, TrC-treated group and Atgli-treated group. LPS (500 ng/mL) was administrated in all groups except the control group. Cells were pretreated with 1 μM Triascin C (MCE, Shanghai, China) or 30 μM Atglistatin (MCE, Shanghai, China) for 60 min and then stimulated with LPS for 24 h. For cell debris-treated experiments, HT22 neuronal cells were used for cellular debris preparation. The cells were maintained in DMEM supplemented with 10% FBS, penicillin, and streptomycin and incubated at 37° C under 5% CO_2_. Cellular debris was produced by repeated freeze–thaw cycles for 3 times as previously described [17]. The cellular debris was then resuspended by sterile PBS, labeled with DiD dye at 37° C for 15 min, and washed by PBS for 3 times.

### Animal and Experimental Model

Eight-week-old male C57/BL6J mice were purchased from the Animal Model Center of Nanjing Medical University (Nanjing, Jiangsu, China). All the mice were housed in specific pathogen-free (SPF) conditions with free access to food and water.

Mice were randomly divided into the vehicle-treated MCAO group and Atglistatin-treated MCAO group. During the operation, 0.25% avertin was used for anesthetization via intraperitoneal injection. The body temperature of mice was kept at 37.0 ± 0.5 ℃. After dissecting the internal carotid artery (ICA) and the middle cerebral artery (MCA), a 6/0 surgical suture was inserted into the MCA through the ICA until the regional cerebral flow decreased to below 30% of the baseline. The process was monitored by laser Doppler flowmetry (Perimed Corporation, Stockholm, Sweden). After 1 h of occlusion, the filament was removed to allow blood reperfusion. The sham-operated mice underwent all these procedures except for the nylon insertion. Atglistatin (200 μM/kg) or the equal volume of vehicle solution were injected into mice intraperitoneally at 24 h, 48 h and 72 h after MCAO.

### Infarct Volume Measurement

The volume of the infarct 3 days after MCAO were measured by Nissl staining. Prepared 20 μm brain sections were stained with 1% cresyl violet for 20–30 min at room temperature. Nissl differentiation, 70% ethanol and 100% ethanol were next used for differentiation. ImageJ software was applied for subsequent analysis. The percentage of the infarct volume was calculated as follows: percentage of the infarct size = (contralateral area − ipsilateral non-infarct area) / (2 × contralateral area) × 100%.

### Neurobehavioral Test

Behavioral tests were conducted to measure the neurological deficits of MCAO mice 24 h/72 h post-surgery. The modified neurological severity score (mNSS), grip strength, and foot fault test were respectively carried out in a two-blinded way. The score of mNSS ranges from 0 to 12 based on the evaluation of motor and balance deficits. A higher score indicates more severe deficits. Grip strength was measured by the grip strength meter (GS3, Bioseb, France). Mice were allowed to grasp the platform of grip strength meter and then pulled straightly in a line. The maximum strength of mouse forepaws was recorded for measurement of grip strength. For foot fault test, prior to the MCAO surgery, mice were trained for 3 days on an elevated grid with a 25 cm width, 40 cm length and 30 cm height. 1-d and 3-d post ischemia, mice were placed on the center of grids for 2 min, which was recorded by a camera. The percentage of failure to step on the grids by contralateral forelimbs were counted manually.

### Immunofluorescent Staining

Mice were anaesthetized and then executed via cardiac perfusion with PBS and 4% paraformaldehyde. Brain tissues were sectioned into 20 μm slices after dehydration. The brain sections were permeabilized in 0.25% Triton X-100 for 20 min, blocked with 2% BSA for 2 h at room temperature, and incubated with primary antibodies at 4 °C overnight. Primary antibodies used included anti-Iba1 (1:500, Abcam, ab5076), anti-Tmem119 (1:500, Synaptic Systems, 400 011), anti-NeuN (1:500, Abcam, ab104224), anti-GFAP (1:500, Bioworld, BS9820M), anti-dMBP (1:200, Millipore, ab5864), anti-CD68 (1:500, Abcam, ab53444). On the next day, the slices were washed 3 times with PBS and incubated with secondary antibodies (Invitrogen, USA) for 2 h in the dark. DAPI (5 g/mL) was used to stain the cell nuclei for 15 min. Finally, images were acquired with a confocal fluorescence microscope (Olympus FV3000, Japan) and analyzed with ImageJ software.

### BODIPY Staining

To detect the accumulation of intracellular neutral lipids in microglia, BODIPY staining were performed together with Immunofluorescent staining. Following the incubation of secondary antibodies, brain slices were incubated with BODIPY staining solution (1:1000 diluted with PBS) at 37 °C for 15 min in the dark and washed with PBS for 3 times.

### Three-Dimensional Reconstruction

As previously described [18, 19], the surface-rendering of Imaris software (Version 9.0.1) was employed for 3D-reconstruction to determine the BODIPY volume inside IBA1^+^ cells, as well as the relationship between degraded MBP and microglia. Confocal Z-stack images were acquired and then processed by the software. Region of interest was selected, and the parameters were set to create surfaces of the chosen channels. The volume of BODIPY signal inside the IBA1^+^ cells was automatically calculated by the software.

### Real-Time Quantitative PCR

Samples from primary microglia were collected. 3 days post MCAO, mice were sacrificed via cervical dislocation after anesthesia. The ischemic hemispheres were then dissected and collected in clean tubes for further experiments. For the sham mice, the tissues in the same hemisphere were isolated. Total RNA of these cells and tissues mentioned above were extracted by TRIzol reagent (Invitrogen) following the manufacturer’s instructions. Afterwards, the RNA was transcribed into cDNA using the PrimeScript RT Reagent Kit (Vazyme, Nanjing, China). Real-time PCR was conducted using a Step One Plus PCR system (Applied Biosystems, Foster City, CA, USA) with 10 μL reaction mixture using a SYBR Green Kit (Applied Biosystems). The corresponding primers were as listed in Table S1.

### Flow Cytometry and Microglia Isolation

Brains were quickly isolated from the Sham/MCAO mice after perfusion with sterile PBS. Ischemic hemispheres were then separated and collected in 1 × HBSS containing HEPES and 25% glucose. For single cell suspension preparation, brain tissues were mechanically grinded and then passed through a 70 μm filter. Cells were stratified by 30–70% Percoll gradient (GE Healthcare BioSciences, USA) and centrifuged at slow acceleration and deceleration at 2,500 rpm for 20 min. Myelin debris on the top layer was discarded, while cells at the interface were carefully collected for further experiments. BODIPY dye (1:10000 dilution) as well as antibodies including anti-CD45 (Biolegend, 103114, 1:1000) and anti-CD11b (Invitrogen, 2513551, 1:500) were used for cell staining. After incubating for 30 min in the dark, cells were washed for three times with 1 × HBSS. After resuspension, CD11b^+^CD45^low^ cells microglia were then isolated by fluorescence activated cell sorter (FACS, BD Biosciences, Carlsbad, CA, USA) and used for further experiments.

### RNA Sequencing

Total RNA was respectively extracted from BODIPY^high^/BODIPY^low^ microglia isolated from ischemic hemispheres in MCAO 3d mice with TRIzol (Invitrogen). Microglia from six hemispheres were pooled into one sample. A NanoDrop 2000 (Thermo Scientific, USA) and Agilent 2100 Bioanalyzer (Agilent, USA) were applied for the assessment of RNA quality. RNA-sequencing transcriptome analysis and downstream analysis were conducted by SEQHEALTH (Wuhan, China). Differentially expressed genes (DEGs) with a |logFC|≥ 1 and P-value of < 0.05 were picked out. Hierarchical cluster analysis of differentially expressed genes (DEGs) was performed to identify the expression patterns of genes in different groups. GO enrichment and KEGG pathway enrichment analysis of DEGs were carried out for further analysis.

### Statistical Analysis

All the data were analyzed using Prism 8.0 software (GraphPad Software, USA). Numerical data are expressed as the mean ± SEM. Student’s t-test was applied to determine the statistical significance between two groups. The statistical significance of data with one factor among multiple groups was evaluated by one-way analysis of variance (ANOVA) followed by Tukey’s post-hoc test. Two-way ANOVA followed by Sidak’s multiple comparisons test was used to test the statistical significance of two factors among multiple groups. P < 0.05 was considered statistically different.

## RESULTS

### LDs Accumulated in Microglia At the Acute Stage of Ischemic Stroke

**Fig. 1:**
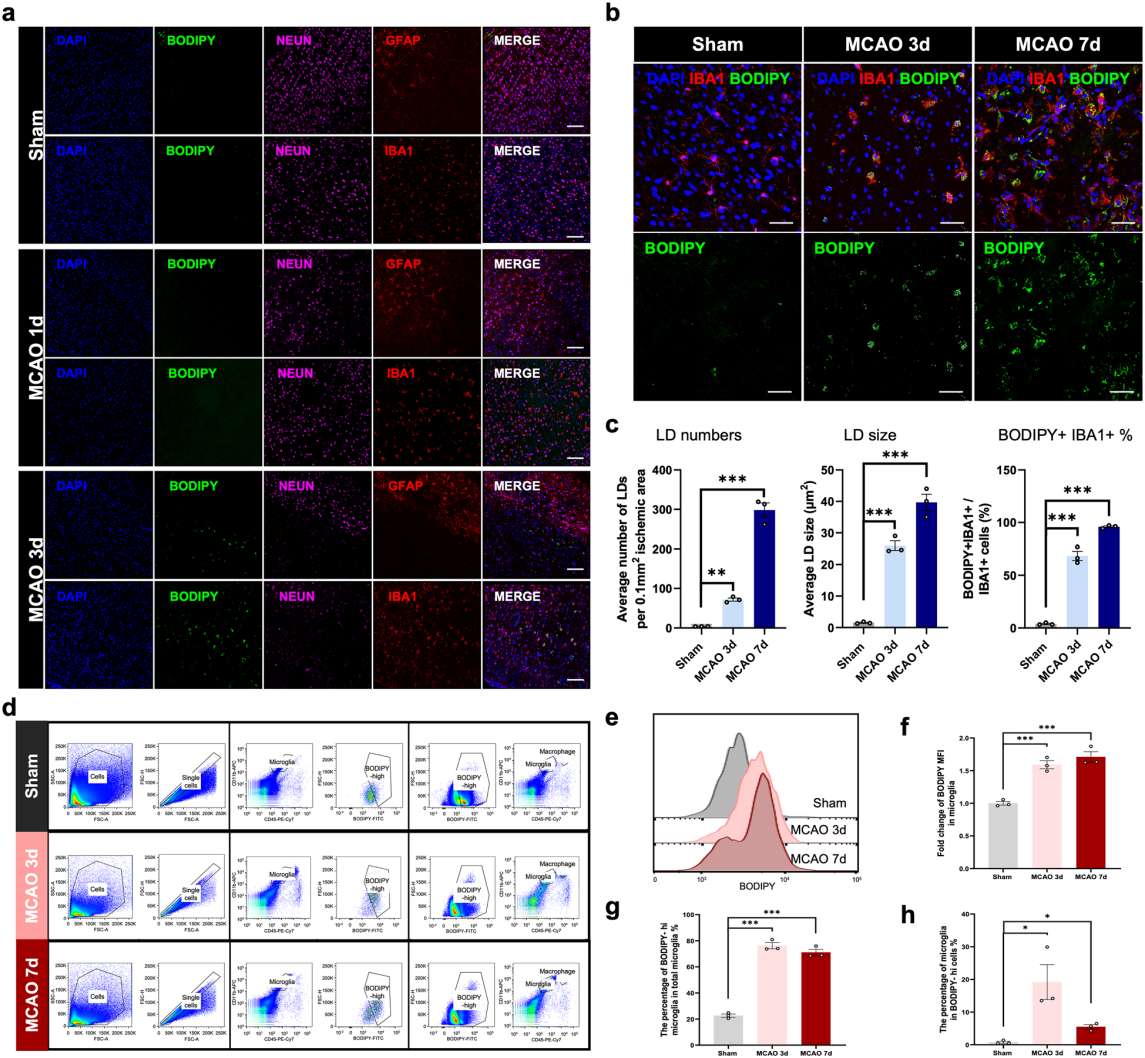
LDs accumulated in microglia after ischemic stroke **a** Representative immunofluorescence images co-staining neuronal-marker (NEUN), astrocyte-marker (GFAP), microglia/macrophage-marker (IBA1) and lipid droplet dye (BODIPY) within the infarct in the sham or MCAO mouse brain sections under confocal observation (scale bar. 100 µm). **b** Representative immunofluorescence images stained for microglia/macrophage-marker (IBA1) and lipid droplet dye (BODIPY) in the infarct regions within brain sections of sham and MCAO (3d and 7d post stroke) mice (scale bar. 50 µm). **c** Quantification of average LD number, LD size and the percentage of BODIPY^+^IBA1^+^ cells in all the IBA1^+^ cells. Data are presented as the mean ± SEM. **P < 0.01, ***P < 0.001, by one-way ANOVA. n = 3/group. **d** Flow cytometry scheme sorting CD11b^+^CD45^low^ microglia from the right hemispheres of sham or MCAO (3d and 7d post stroke) mice. BODIPY^high^ microglia were further sorted from microglia. CD11b^+^CD45^low^ microglia were sorted from BODIPY^high^ cells at the same time. **e** Representative flow cytometry histogram showing the mean fluorescence intensity (MFI) of BODIPY in microglia isolated from sham or MCAO mice. **f-h** Quantitation of BODIPY MFI in microglia sorted from right hemispheres in sham or MCAO mice (3d and 7d post the surgery) **f**. The percentage of BODIPY^high^ microglia in total microglia **g** and the percentage of microglia among BODIPY^high^ cells **h** were quantified respectively. Data are presented as the mean ± SEM. *P < 0.05, ***P < 0.001, by one-way ANOVA. n = 3/group.

To determine whether LDs accumulated after ischemic stroke, we used BODIPY, a neutral lipid dye, for LDs detection. At 3 days post-middle cerebral artery occlusion (MCAO) surgery, we observed significant LDs accumulation within the ischemic lesion. Whereas, LDs formation was undetected at 1 day after MCAO. In order to determine the cell type that produced LDs after ischemic stroke, we co-stained astrocyte-specific marker GFAP, neuron-specific marker NeuN and microglia/macrophage marker IBA1 with BODIPY. Strikingly, LDs were extremely immersed in IBA1^+^ cells in the lesion site and we barely detected LDs in neurons and astrocytes at the acute stage of ischemic stroke (Fig. 1a). To further illustrate the temporal alterations of LDs in microglia/macrophage during the acute stage of cerebral ischemia, we performed immunostaining on sham or MCAO brain sections at both 3-day and 7-day post stroke. In the infarct core, the percentage of BODIPY^+^IBA^+^ cells in IBA1^+^ cells significantly increased during the acute stage of stroke. LDs tended to increase in both their number and size in IBA1^+^ cells at MCAO 7d compared with MCAO 3d (Fig. 1b, c). For further verification, we conducted 3D reconstruction to show the co-localization of LD and IBA1^+^ cells. The results showed that the volume of LDs significantly increased upon acute ischemia (Fig. S1). Moreover, to determine whether LDs remained or disappeared during the chronic phase of stroke, we further performed BODIPY staining at 14 days and 30 days post-stroke. As shown in Fig. S2, LDs remained at 14 days after MCAO, but nearly vanished at 30 days post-stroke. Therefore, LDs accumulated in microglia at the acute stage of ischemic stroke.

To verify these findings, we next performed flow cytometry by isolating the ipsilateral hemispheres of sham or MCAO mice (Fig. 1d). Consistent with immunofluorescence results, there was a significant increase in the mean fluorescence intensity (MFI) of BODIPY in CD11b^+^CD45^low^ microglia after cerebral ischemia (Fig. 1e, f). More than half of microglia showed LDs accumulation at 3-d and 7-d after ischemic insult (Fig. 1g). In addition, the percentage of microglia in BODIPY^high^ cells was highest at 3 days post ischemia, highlighting the role of stroke-associated foamy microglia at the acute stage of ischemic stroke (Fig. 1h).

### Foamy Microglia With Unique Transcriptomes Were Associated With Inflammation and Phagocytosis

**Fig. 2:**
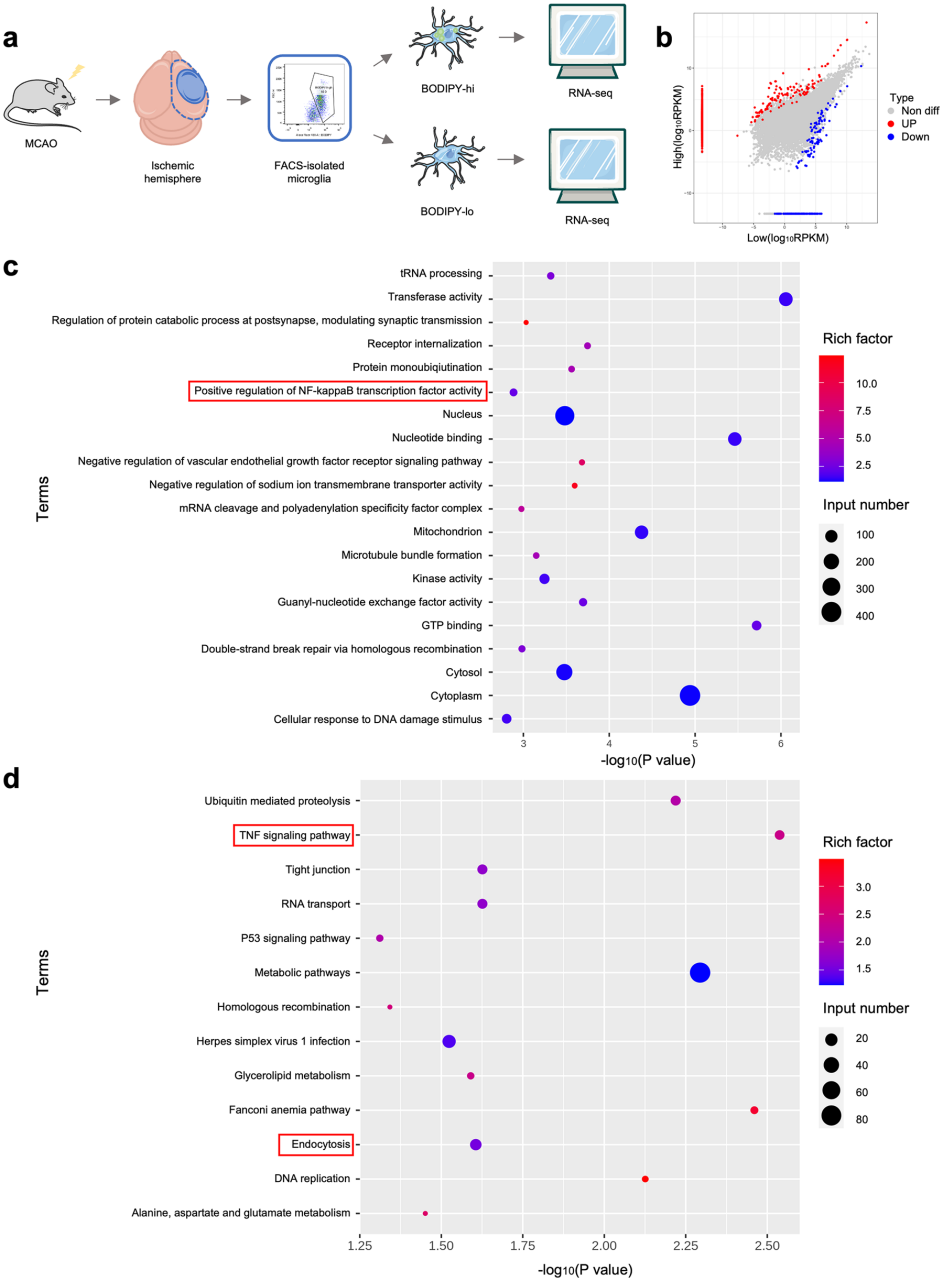
Foamy microglia with unique transcriptomes were associated with inflammation and phagocytosis. **a** Experimental schematic of RNA-sequencing (RNA-seq) in fluorescently activated cell sorting (FACS)-isolated BODIPY-high / BODIPY-low microglia from ischemic hemispheres of MCAO 3d mice. Six hemispheres were pooled into one sample. n = 3/group. **b** Scatter plot showing differentially expressed genes (DEGs) between BODIPY-high microglia and BODIPY-low microglia. |logFC (fold change)|> 1, p value < 0.05. **c**, **d** GO **c** and KEGG **d** enrichment analysis of DEGs between BODIPY-high microglia and BODIPY-low microglia.

To identify the transcriptomic characteristics of stroke-associated foamy microglia, we isolated CD11b^+^CD45^low^ microglia from ischemic hemispheres in MCAO 3d mice. Based on the MFI of BODIPY, we further divided CD11b^+^CD45^low^ microglia into BODIPY^high^ microglia and BODIPY^low^ microglia, which were subsequently performed RNA-sequencing (RNA-seq) (Fig. 2a). A total of 1184 genes were differentially expressed between BODIPY^high^ microglia and BODIPY^low^ microglia, with 968 genes upregulated and 216 genes downregulated (Fig. 2b). Gene ontology (GO) and Kyoto encyclopedia of genes and genomes (KEGG) enrichment analysis of differentially-expressed genes (DEGs) were performed. As revealed in the Fig. 2c and d, inflammation-related pathways like ‘positive regulation of NF-kappaB transcription factor activity’ and ‘TNF signaling pathway’ were enriched in BODIPY^high^ microglia. ‘Endocytosis’ was also significantly enriched in foamy microglia, indicating a close relationship between stroke-associated foamy microglia and phagocytosis (Fig. 2d).

### Inflammatory Microenvironment and Debris Phagocytosis Drove LDs Formation in Microglia

**Fig. 3:**
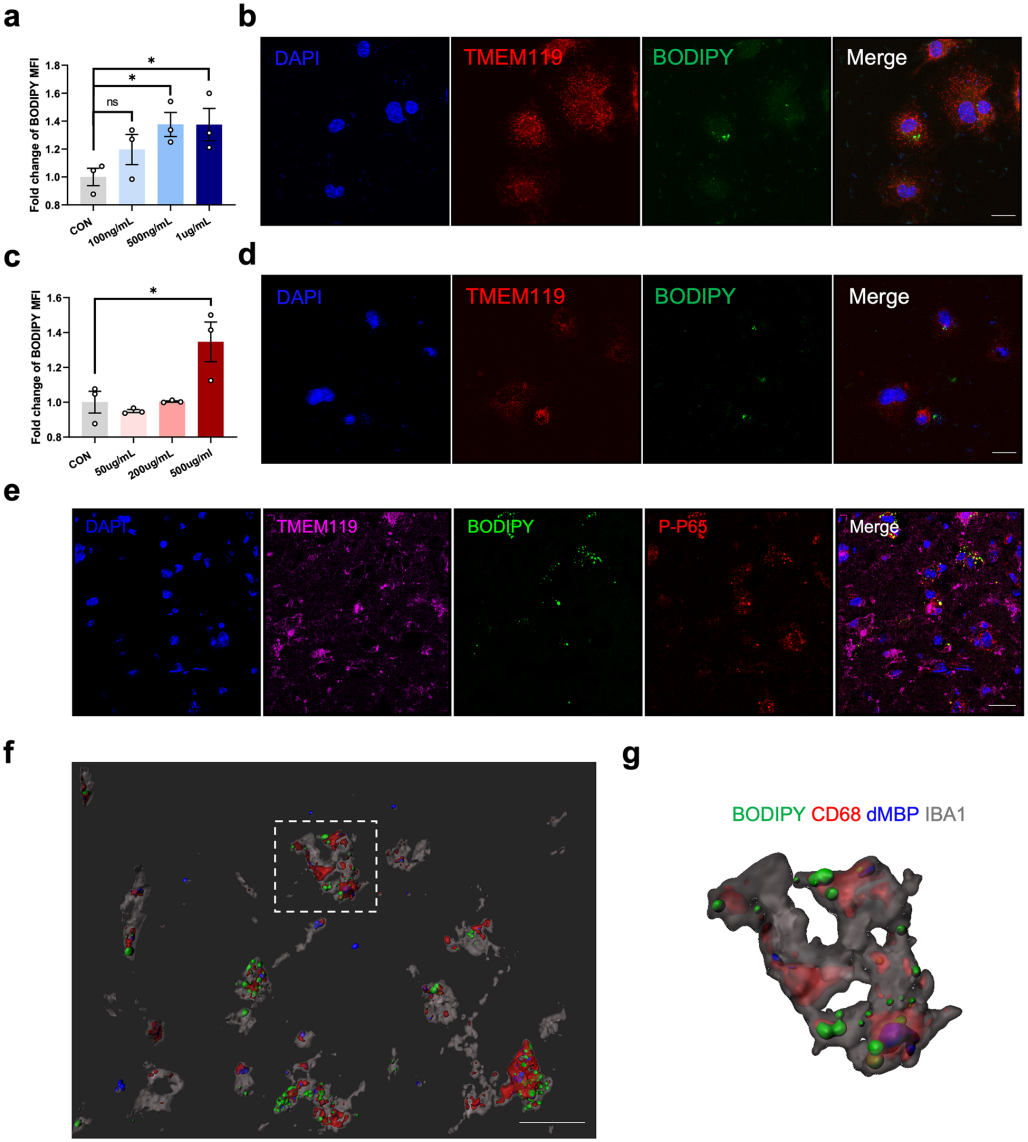
Inflammatory microenvironment and debris phagocytosis drove LD formation in microglia. **a **Quantification of BODIPY MFI, measured by flow cytometry, in primary microglia after LPS treatment at different concentrations (100 ng/mL, 500 ng/mL and 1 μg/mL) for 24 h. Data are presented as the mean ± SEM. *P < 0.05, by one-way ANOVA. n = 3/group. **b **Representative immunofluorescence images stained for BODIPY (lipid droplet dye) and TMEM119 (microglia marker) in primary microglia after LPS treatment (500 ng/mL, 24 h) (scale bar. 20 µm). **c** Quantification of BODIPY MFI in primary microglia after cellular debris treatment at different concentrations (50 μg/mL, 200 μg/mL and 500 μg/mL) for 24 h by flow cytometry. Data are presented as the mean ± SEM. *P < 0.05, by one-way ANOVA. n = 3/group. **d** Representative immunofluorescence images stained for BODIPY (lipid droplet dye) and TMEM119 (microglia marker) in primary microglia after cellular debris treatment (500 μg/mL, 24 h) (scale bar. 20 µm). **e** Representative immunofluorescence images showing the expression of phospho-NF-κB p65 (p-p65) within BODIPY^+^TMEM119^+^ cells in the infarct from MCAO 3d mice brain sections (scale bar. 50 µm). **f**, **g** 3D reconstruction of the existence of degraded MBP (dMBP) within CD68^+^BODIPY^+^IBA1^+^ cells **f** (scale bar. 20 µm). The right panel was the magnification of the selected cells **g**.

Recent findings positioned LPS, an exogeneous TLR4 ligand, as the key upstream regulator of LDs formation in microglia [7–9]. To confirm whether inflammation had the capacity of triggering LDs accumulation, we treated primary microglia with different concentrations of LPS. A concentration-dependent increase was then observed in BODIPY MFI in primary microglia upon LPS stimulation. As shown in Fig. 3a, b, obvious LDs formation occurred in primary microglia after LPS treatment for 24 h (Fig. 3a, b). We subsequently examined the expression of phospho-NF-κB p65 (p-p65), the downstream responder of TLR4, in foamy microglia after stroke. As predicted, the co-localization of p-p65 and BODIPY^+^TMEM119^+^ microglia was observed in the ischemic core (Fig. 3e). Therefore, post-stroke inflammatory microenvironment might drove LDs formation in microglia.

Additionally, large amounts of cellular and myelin debris generated in response to ischemic insult could be engulfed by activated microglia. Lipid-rich cellular membrane and myelin debris probably lead to LDs formation [10]. We wondered whether this was another potential trigger for LDs induction. To test this hypothesis, we prepared cellular debris by repeated freeze–thaw of HT22 cell line. Then, we stimulated primary microglia with different concentrations of cellular debris. Flow cytometry and immunostaining were performed to examine the MFI of BODIPY and visible LDs formation in primary microglia treated with cellular debris (Fig. 3c, d). As expected, cellular debris also successfully induced LDs accumulation in microglia. Furthermore, almost all the foamy microglia were CD68-immunopositive. Three-dimensional (3D) reconstruction was performed to reveal that degraded MBP (dMBP) also accumulated in CD68^+^BODIPY^+^IBA1^+^ cells, suggesting that microglial phagocytosis of debris at least partly contributed to LDs accumulation (Fig. 3f, g). Therefore, these data revealed an important role of inflammatory stimulus and cellular/myelin debris in the LDs formation in microglia after ischemic stroke.

### LDs-Reduction Exacerbated Microglia-mediated Pro-inflammatory Responses

**Fig. 4:**
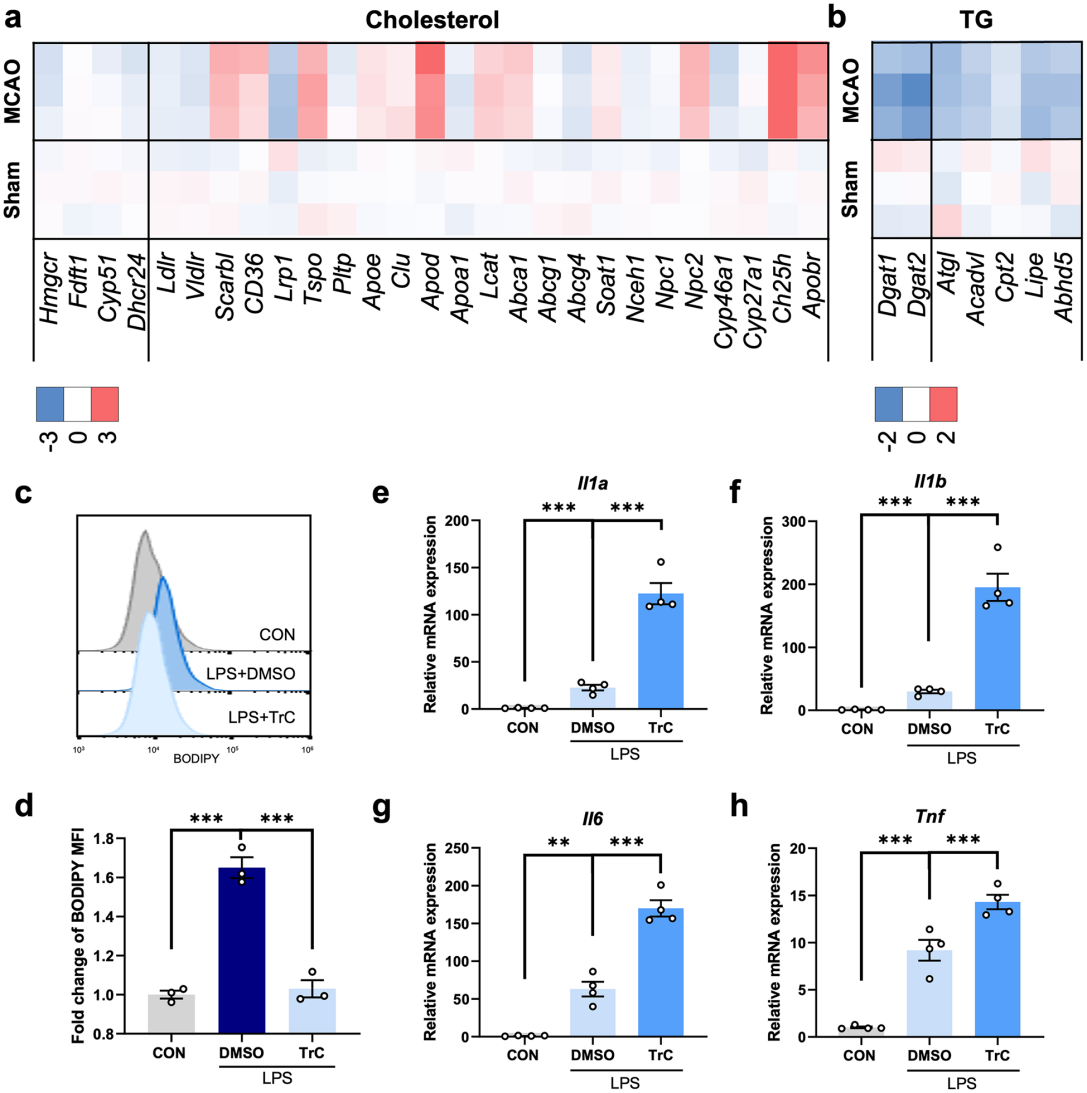
LDs-reduction exacerbated microglia-mediated pro-inflammatory responses. **a**, **b** Heatmaps showing the expression level of cholesterol **a**/ triacylglycerol (TG) **b** synthesis and metabolism-associated genes in the right hemisphere from sham or MCAO 3d mice by real-time PCR analysis. Genes in the left compartment were responsible for cholesterol/TG synthesis, while genes in the right compartment were lipid metabolism-related. n = 3/group. **c**, **d** Representative flow cytometry histogram showing the mean fluorescence intensity (MFI) of BODIPY in primary microglia after LPS stimulation (500 ng/mL, 24 h) with or without Triascin C (TrC) pretreatment (1 μM, 1 h) **c**. The BODIPY MFI was quantified **d**. Data are presented as mean ± SEM. ***P < 0.001, by one-way ANOVA. n = 3/group. **e**–**h** Real-time PCR analysis of the expression of pro-inflammatory factors (*Il1a, Il1b, Il6* and *Tnf*) in primary microglia upon LPS stimulation (500 ng/mL, 24 h) with or without TrC pretreatment (1 μM, 1 h). Data are presented as mean ± SEM. **P < 0.01, ***P < 0.001, by one-way ANOVA. n = 4 biological independent samples.

Furthermore, the core of neutral lipids was composed of di/tricylglycerols, sterol esters, et al. Considering the difficulty of isolating *in vivo* microglial LDs for lipid analysis, we examined the expression level of cholesterol/TG synthesis and metabolism-related genes using quantitative PCR analysis instead of targeted metabolomics to speculate the main lipid content of microglial LDs. Interestingly, the expression level of genes associated with either cholesterol or TG synthesis decreased in the ischemic hemispheres compared with control hemispheres. The expression of cholesterol metabolism-related genes, however, upregulated at 3 days after MCAO, indicating decreased sterol level following stroke. TG metabolism-related genes were, instead, significantly downregulated (Fig. 4a, b). These results indicated that the main lipid content of LDs was probably TG instead of sterol.

To gain insight into the function of LDs in inflammation modulation, we treated LPS-stimulated primary microglia with Triacsin C (TrC), an inhibitor of Acyl-CoA synthetase (ACSL) reported to efficiently downregulate TG levels and reduce LDs formation. By flow cytometry, we detected a significant decrease of BODIPY MFI in TrC-treated group, verifying the capacity of TrC in the inhibition LDs-formation (Fig. 4c, d). Then, we measured the expression level of several classical pro-inflammatory cytokines among different groups by using qPCR analysis. Compared with DMSO-treated microglia after LPS stimulation, *Il1α*, *Il1β*, *Il6* and *Tnf* were remarkably upregulated in the TrC-treated microglia, suggesting the potential anti-inflammatory properties of LDs (Fig. 4e-h). The same experiments were performed in the cellular debris group and the results were consistent with those of LPS-treated cells (Fig. S3).

### ATGL Inhibitor Prevented Inflammation and Exhibited Neuroprotective Effects

**Fig. 5:**
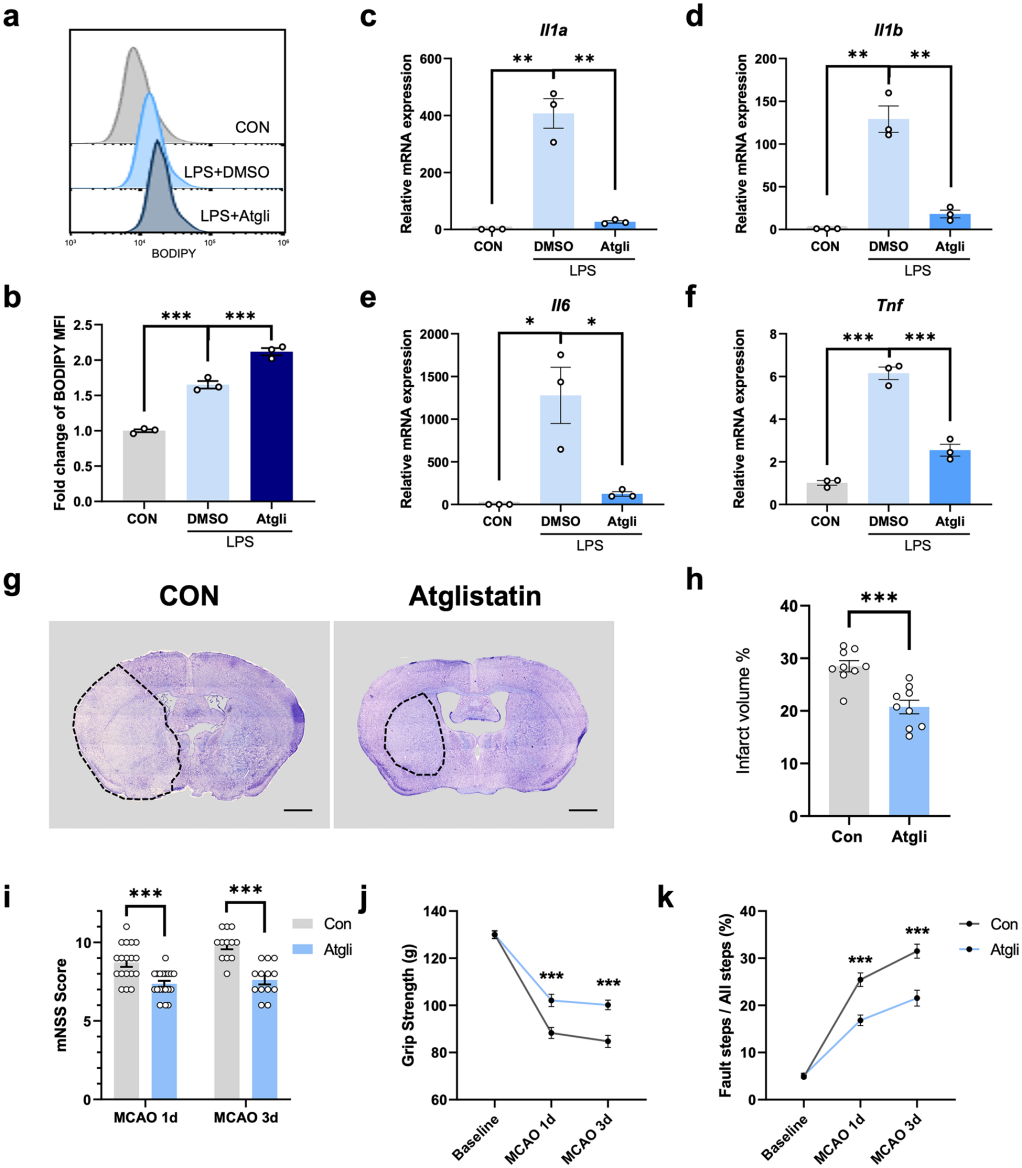
ATGL inhibitor prevented inflammation and exerted neuroprotective effects. **a**, **b** Representative flow cytometry histogram displaying the BODIPY MFI in primary microglia after LPS stimulation (500 ng/mL, 24 h) with or without Atglistatin (Atgli) pretreatment (30 μM, 1 h) **a**. The BODIPY MFI was quantified **b**. Data are presented as mean ± SEM. ***P < 0.001, by one-way ANOVA. n = 3/group. (**c**-**f**) Real-time PCR analysis of the mRNA level of pro-inflammatory cytokines (*Il1a, Il1b, Il6* and *Tnf*) in primary microglia upon LPS stimulation (500 ng/mL, 24 h) with or without Atglistatin treatment (30 μM, pretreatment for 1 h). Data are presented as mean ± SEM. *P < 0.05, **P < 0.01, ***P < 0.001, by one-way ANOVA. n = 3/group. **g** Representative nissl staining images of brain sections from MCAO 3d mice administered with vehicle or Atglistatin respectively (scale bar. 1000 µm). **h** The quantification of infarct volume measured by nissl staining of MCAO 3d mice in control and Atglistatin group. ***P < 0.001, by Student’s t-test. n = 9/group. **i**-**k** Neurological performance of vehicle-treated and Atglistatin-treated mice both at 1 day and 3 days post the MCAO surgery, including mNSS score **i**, grip strength **j** and foot fault **k**. ***P < 0.001, by two-way ANOVA. n = 12–20 /group.

Based on our results, the main content of LDs contained in stroke-associated foamy microglia was probably TG, and downregulation of LDs through TrC could remarkably increase microglial production of pro-inflammatory cytokines. Considering that strengthened lipolysis in LDs was reported to promote inflammatory mediators production, we wondered whether targeting LDs by preventing lipolysis would exhibit inflammation-alleviating effects in microglia and thus exert neuroprotective effects on MCAO mice. Adipose triglyceride lipase (ATGL) was accepted to be responsible for the lipolysis of TG, and Atglistatin was a specific ATGL inhibitor. Several publications have indicated the potential capacity of Atglistatin in the modulation of inflammation [20]. Thus, we chose Atglistatin for further investigation. We first performed the flow cytometry to measure the BODIPY MFI in Atglistatin-treated or untreated primary microglia, verifying that Atglistatin indeed promoted LDs-formation in microglia. As shown in Fig. 5a, b, the MFI of BODIPY significantly increased after Atglistatin pretreatment (Fig. 5a, b). The expression of a series of inflammatory cytokines also strikingly decreased in the Atglistatin-treated group by qPCR analysis (Fig. 5c-f).

We then hypothesized that ATGL inhibitor could probably be neuroprotective via targeting LDs in microglia during the progression of ischemic stroke. Therefore, we administrated MCAO mice with Atglistatin or vehicle and then measured neurological performance at 1-d and 3-d post ischemia. Atglistatin significantly decreased the infarct volume 3 days post MCAO surgery (Fig. 5g, h). Meanwhile, significantly-improved neurological performance with lower mNSS score, decreased percentage of foot fault and stronger grip strength was also observed in the Atglistatin-treated group (Fig. 5i-k). Based on these data, we concluded that Atglistatin could ameliorate ischemic injury and neurological deficits, highlighting that the pharmacological modulation of LDs-formation in microglia might become a novel therapeutic strategy at the acute stage of ischemic stroke.

## DISCUSSION

Currently, the cell type with LDs accumulation, the factors triggering LDs formation and its exact function during the pathological processes of ischemic stroke are not fully elucidated. Here, we found that LDs mainly accumulated in microglia at the acute stage of ischemic stroke. The activation of TLR4 and phagocytosis of myelin/cellular debris might contributed to LDs formation in microglia. TG might be the main content of the neutral lipid core within LDs. By using an ACSL inhibitor TrC, we surprisingly found the potential anti-inflammatory properties of stroke-associated microglial LDs and further confirmed the neuroprotective effects of an ATGL inhibitor Atglistatin in ischemic stroke.

In our study, we observed conspicuous presence of LDs in microglia 3-d after cerebral ischemia, while Arbaizar-Rovirosa M et al. identified lipid-laden microglia 1-d post MCAO surgery [21]. This difference could be explained by the different age of mice used and approaches applied for LDs identification. Consistent with our findings, both studies suggested that LDs formation in microglia significantly increased at the acute stage of ischemic stroke. To determine whether LDs remained or disappeared during the chronic phase of stroke, we further performed BODIPY staining at 14 days and 30 days post-stroke. As shown in Fig. S2, LDs remained at 14 days after MCAO, but nearly vanished at 30 days post-stroke. Compared with abundant LDs accumulation in the acute phase, it was reasonable to speculate that LDs were gradually cleared during the chronic phase of stroke. Previous studies have identified that lipolysis and lipophagy were the two main ways of LDs turnover. During lipolysis, lipases were responsible for the hydrolysis of neutral lipids, wherein ATGL was the first and rate-limiting enzyme [22]. LDs could also be engulfed by autophagosomes and then delivered to lysosomes, being hydrolyzed into free fatty acids [23, 24]. Novel lipophagy candidate factors (*Hmgb1*, *Hmgb2*, *Hspa5*, *Scarb2*, *Ube2g2*, *Aup1*, *etc*.) have been recently identified to allow selective manipulation of lipophagy in foamy macrophages [25]. Whereas, how cells were directed to lipolysis or lipophagy for clearance of LDs, and how these two routes interacted were still unknown. It was speculated that extracellular nutritional status and intracellular lipids themselves might influenced these processes [24, 26]. Interestingly, we also observed several LDs began to be outside IBA1^+^ cells at 14 days after stroke. Thus, it was assumed that LDs became to accumulate in other cell types or transported from microglia to adjacent cells at the chronic stage of stroke. Previous studies reported that the transfer of LDs from neurons to astrocytes was mediated by ApoE-positive lipoprotein particles [27]. However, the mechanisms underlying the LDs transfer from microglia to other glial cells or neurons remains poorly understood.

Additionally, immunostaining indicated that LDs were more and larger at MCAO 7d than MCAO 3d, while there was no significant increase in microglial BODIPY MFI at 7-d post ischemia compared with MCAO-3d in flow cytometry assay. Since IBA1 could be expressed by both microglia and macrophage, macrophage might infiltrated the brain through disrupted blood brain barrier (BBB) and were accumulated with LDs after ischemic stroke, thus contributing to exaggerated LDs formation in IBA1^+^ cells in immunofluorescent images. However, only about twenty percent of BODIPY-high cells are microglia. We speculated that infiltrating macrophages, as well as oligodendrocytes which produced neutral lipid-containing myelin, could accounted for part of BODIPY-high cells [28].

Although several studies pointed to a critical role for LDs in the functional modulation of microglia, the mechanism of LDs formation remained poorly-identified. After stroke, large amount of tissue debris was generated and microglia exhibited enhanced phagocytosis [29]. Using single-cell transcriptomes, stroke-associated myeloid cells (SAMC) were discovered and exhibited enhanced lipid metabolism and phagocytosis, indicating a potential role of microglia in the modulation of stroke outcome through clearance of lipid-rich tissue debris. In addition, BODIPY was preferentially positive in cells expressing SAMC markers within the infarct, suggesting a close relationship between enhanced phagocytosis and LDs accumulation [30]. In MBP-KO mice, LDs still occurred after spinal cord injury (SCI), indicating that myelin was not the only source of lipid that inducing the formation of foamy macrophages [31]. Our data revealed that both inflammatory activation and phagocytosis of cellular/myelin debris could promote the generation of LDs in microglia. It has been identified that ApoA-I binding protein (AIBP) could bind to activated microglia via its N-terminal domain which was the binding site for TLR4, reduced accumulation of LDs in microglia within the spinal cord and exerted anti-inflammatory effects in a model of chemotherapy-induced peripheral neuropathy [32]. Thus, TLR4 activation in microglia also at least partly accounted for the formation of LDs. Besides inflammatory activation and phagocytosis of cellular/myelin debris, type I IFN was also reported to play a vital role in LDs induction for the abrogated LDs generation in Stat1^−/−^ mice defective in the transduction of IFN signal after stroke [21]. Evidenced by several studies, TREM2, a lipid-sensing receptor, played a pivotal role in the accumulation of LDs. By transplanting isogenic iPSC-derived human microglia with TREM2 R47H mutation or wild type into a chimeric mouse model of AD. Claes, C. et al. found that LDs were significantly decreased in TREM2-R47H xenografted microglia *in vivo* [15]. Moreover, there was a significant decrease in foamy microglia with LDs in TREM2-deficient mice. Injection of PBA, an chaperone reducing ER stress, could restore LDs formation among IBA1^+^ cells within demyelinated lesions in TREM2-KO mice [10]. Apolipoprotein E4 (APOE4), a genetic risk factor for AD, was also suggested to result in LDs abundance in microglia [33]. Autophagy could also influence LDs formation. Atg7 was an important mediator of autophagosome biogenesis. Microglia deficient in Atg7 showed increased LDs accumulation and cytokine production, which could be rescued by enhanced lipid efflux [34]. Therefore, LDs could be induced by distinct factors among different pathological processes.

LDs has been regarded as a temporary repository for neutral lipid, controlling the spatiotemporally coordinated release of inflammatory lipid mediators [35]. These released lipid mediators could directly or indirectly regulate the PPAR or NF-κB signaling pathway, modulating the expression of inflammation genes [5, 36, 37]. Therefore, by sequestration of lipids from their bioactive pool, LDs reduced the availability of lipid mediators necessary for the activation of specific signaling pathways [38, 39]. Disruption of LDs biosynthesis was demonstrated to activate the NF-κB transcription factor [40]. Our study also provided evidence that inhibition of LDs synthesis promoted the expression of pro-inflammatory cytokines in microglia as shown in Fig. 4e-h. In addition, the degradation of LDs by lipolysis could promote pro-inflammatory responses. Adipose triglyceride lipase (ATGL) and hormone-sensitive lipase (HSL) were the two main lipases of lipolysis. HSL-mediated lipolysis could promote the production of cyclooxygenase-2 and prostaglandin E2 (PGE2) [41]. HSL-dependent sphingosine kinase 1 and JNK signaling activation could also stimulated β-adrenergic signaling which led to the elevated expression of pro-inflammatory genes like IL-6 [42, 43]. Using ATGL-deficient cells or pharmacological inhibitors, several studies indicated that prevention of lipolysis could attenuate the inflammatory responses in immune cells by reducing the ATP supply or the fatty acid precursors for inflammatory mediators production [44–46]. Upregulation of hypoxia-inducible lipid droplet–associated (HILPDA) protein was also capable of suppressing ATGL-mediated lipolysis and restoring LD formation in LPS-activated macrophages, thereby reducing the production of PGE2 and IL-6 [47]. Consistent with these publications, our experiments suggested potential anti-inflammatory properties of LDs in LPS-treated microglia via lipolysis inhibition by Atglistatin.

Based on our results, LDs were able to attenuate pro-inflammatory responses of LPS-stimulated microglia. Whereas, several studies also reported detrimental roles of LDs among other pathological processes. In the ageing brain, LD-accumulating microglia showed increased expression level of pro-inflammatory genes [7–9]. Glial LDs accumulation was also reported to be a promotor of neurodegeneration [3, 4]. As the well-known risk gene for sporadic AD, APOE4 could induce LDs accumulation which impaired microglial surveillance of neuronal activity [13]. Although these findings seemed to be contradictory to our findings, there were several points which might explained the inconsistencies. Firstly, microglial LDs in our study was induced by acute, robust inflammatory microenvironment after ischemia or LPS treatment, where TLR4 played a pivotal role [48]. While ageing, AD and other neurodegenerative diseases were characterized by chronic pathological processes, which were different from ischemic stroke. Thus, it was reasonable to speculate that LDs triggered by different stimulus in different diseases had distinct roles. Moreover, LDs could be beneficial at the early stage but become detrimental during the late phase in the same disease, indicating a temporal-specific role of LDs as well [3]. Additionally, LDs was heterogeneous in their structure, composition and distribution. LDs induced by different factors was equipped with distinct protein and lipid composition, which had a large impact on its function and fate [49, 50]. Proteomic studies revealed a myriad of LD-related proteins in different tissue and cell types which could be simply classified into nine categories with distinct roles in LD-turnover and LD-associated pathologies [51, 52]. Therefore, the effects of LDs depended on different stimulus, pathological phases, lipid and protein compositions, cell and tissue types. Our study mainly focused on the function of LDs in microglia at the acute stage of ischemic stroke. However, the composition of microglial LDs after ischemic insult remains unknown. A proteomic or lipidomic study is needed to explore the mechanisms underlying the anti-inflammatory capacity of microglial LDs in acute ischemic stroke.

In our study, we mainly focused on the capacity of LDs in inflammation modulation and proposed that LD acted as a stable organelle to store inert fat and maintained the anti-inflammatory properties of microglia. Additionally, SwissADME revealed that Atglistatin had good BBB permeability, suggesting a potential application of Atglistatin in stroke treatment (Table. S2). Based on our results, neurological performance of MCAO mice was significantly improved after Atglistatin treatment, showing a neuroprotective role of LDs at the acute stage of cerebral ischemia. However, there was no denying that pharmacological manipulation inevitably affected other genes or pathways irrelevant to lipid metabolism. Accurate investigation should be carried out by genetic manipulation of LDs within microglia at the acute stage of ischemic stroke. Mice strains with microglia-specific knock-out of key enzymes responsible for lipid biogenesis or lipolysis would be more reliable to investigate the precise functions of microglial LDs after stroke.

In summary, we identified microglial LDs accumulation at the acute stage of ischemic stroke. Both inflammatory activation and phagocytosis of tissue debris contributed to increased LDs formation in microglia. Through specific inhibition of LDs biogenesis or lipolysis, we indicated that microglial LDs exhibited protective properties by preventing inflammatory cytokines production in primary microglia. Additionally, ATGL inhibitor remarkably improved the neurological performance of MCAO mice through upregulation of LDs. Collectively, we identified the anti-inflammatory and neuroprotective role of Atglistatin by regulating microglial LDs at the acute stage of ischemic stroke, uncovering new therapeutic options for stroke treatment.

### Supplementary Information

Below is the link to the electronic supplementary material.

Supplementary file1 (TIF 794 KB)

Supplementary file2 (TIF 18804 KB)

Supplementary file3 (TIF 479 KB)

Supplementary file4 (XLSX 14 KB)

Supplementary file5 (CSV 1 KB)

## Data Availability

Experimental data supporting this study can be provided upon request.
