# Supplementary figures and images for: Pharmacological Upregulation of Microglial Lipid Droplet Alleviates Neuroinflammation and Acute Ischemic Brain Injury
Source: Inflammation. 2023 Jul 14;46(5):1832–48. doi: 10.1007/s10753-023-01844-z (PMC10567859; doi:10.1007/s10753-023-01844-z)

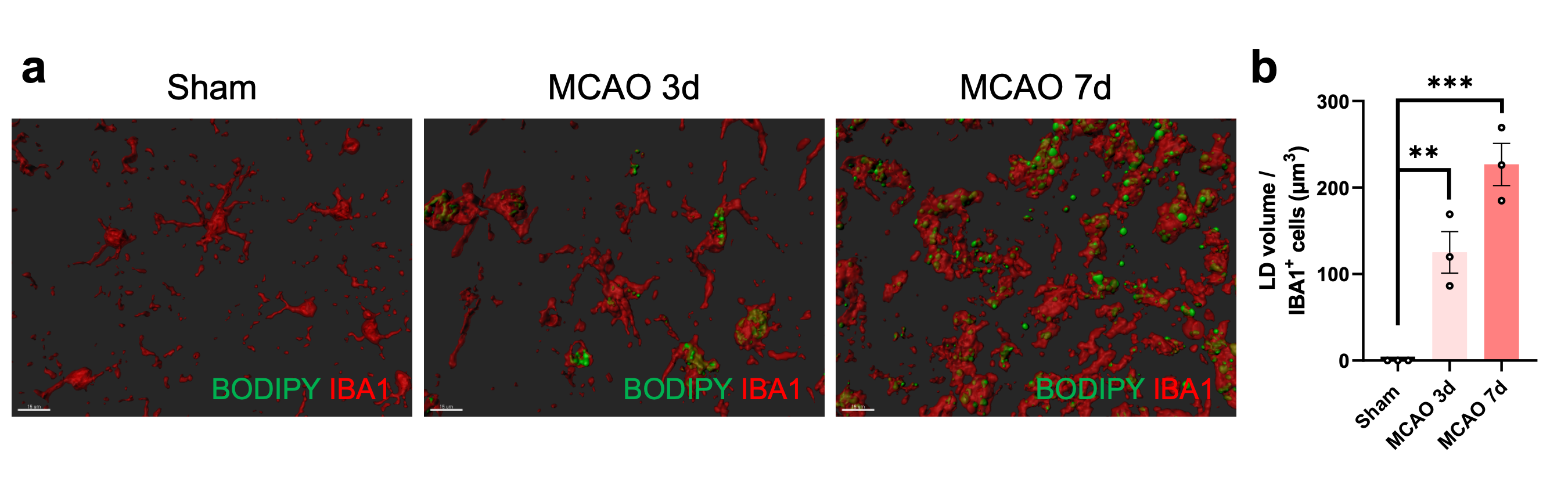

Supplement: Supplementary file 1 — Supplementary file1 (TIF 794 KB) [file 10753_2023_1844_MOESM1_ESM.tif]

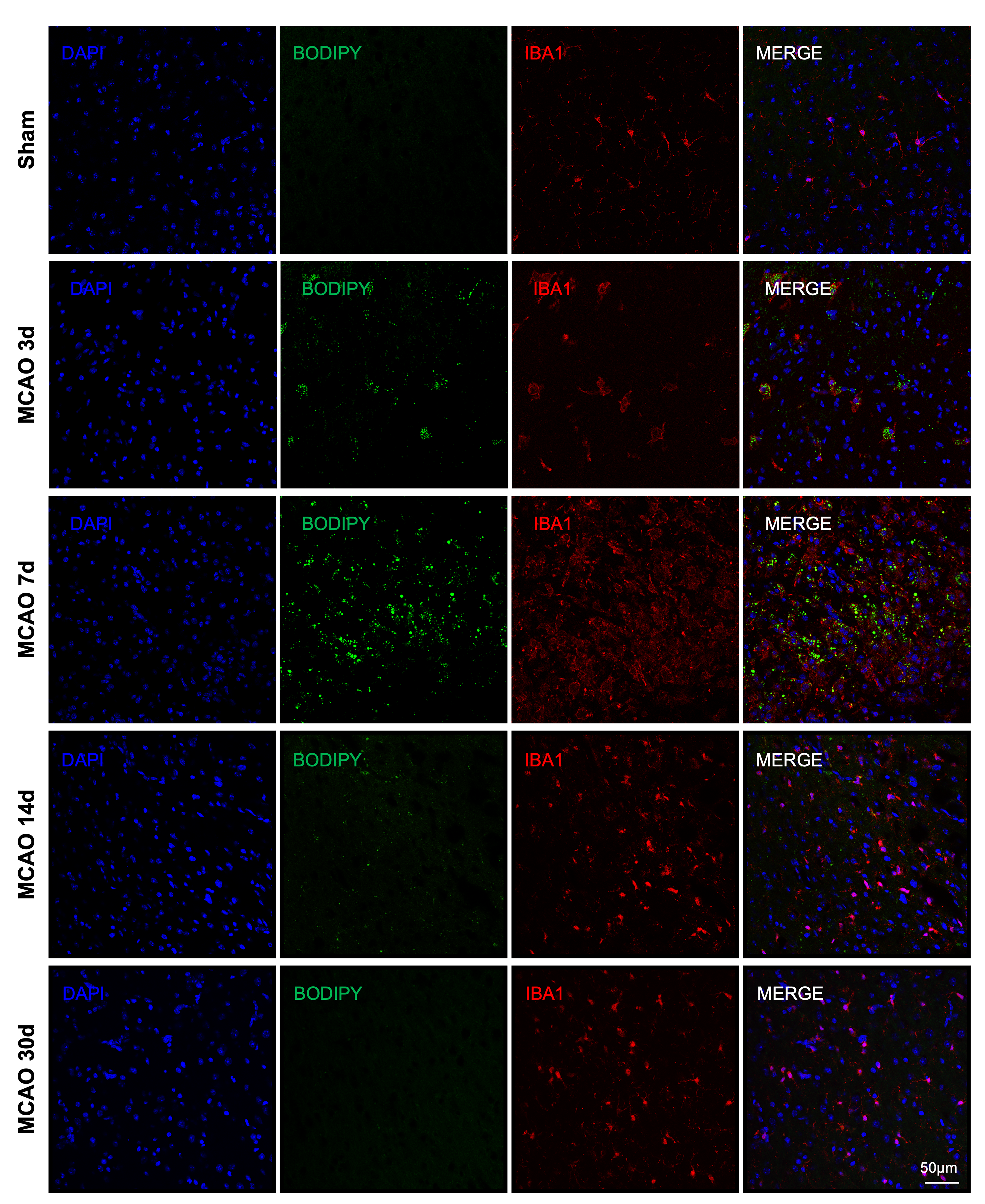

Supplement: Supplementary file 2 — Supplementary file2 (TIF 18804 KB) [file 10753_2023_1844_MOESM2_ESM.tif]

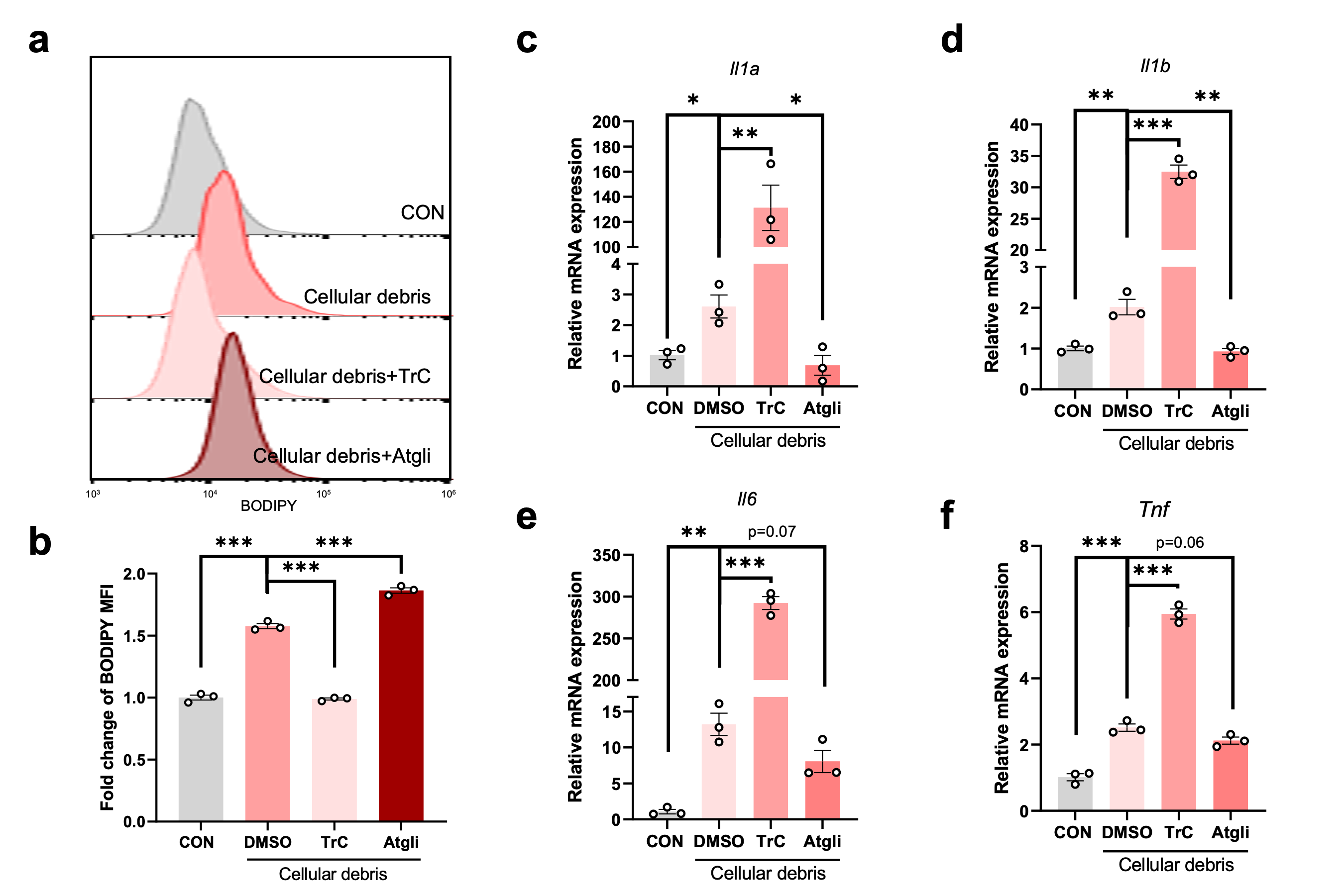

Supplement: Supplementary file 3 — Supplementary file3 (TIF 479 KB) [file 10753_2023_1844_MOESM3_ESM.tif]
